# “MAMA’s is like a second mom:” Client and Staff Experiences in a Comprehensive Social Risk Care Management Program Within a Perinatal Medical Home

**DOI:** 10.1007/s10995-024-03896-5

**Published:** 2024-01-31

**Authors:** Kasee Houston, Flor Arellano, Helia Imany-Shakibai, Ashaki Jackson, Erin Saleeby, Rebecca Dudovitz, Adam Schickedanz

**Affiliations:** 1 Southern California Permanente Medical Group, Department of Neonatal-Perinatal Medicine, Kaiser Permanente Riverside Medical Center, 10800 Magnolia Ave, Riverside, CA 92505 USA; 2 Department of Pediatrics, David Geffen School of Medicine at UCLA, 10833 Le Conte Ave, Los Angeles, CA 90095 USA; 3 Los Angeles County Department of Health Services, 313 N Figueroa St., Los Angeles, CA 90012 USA; 4 Harbor UCLA Medical Center, 1000 W Carson St., Torrance, CA 90502 USA; 5 Department of Health Policy and Management, UCLA Fielding School of Public Health, Los Angeles, CA USA

**Keywords:** Preterm birth, Social determinants of health, Racism, Discrimination, COVID-19

## Abstract

**Introduction:**

Addressing persistent racial inequities in preterm birth requires innovative health care approaches. The Los Angeles County Maternity Assessment and Management Access Service Synergy Neighborhood program (MAMA’s) is a perinatal medical home program designed to alleviate the impacts of chronic stress by addressing social determinants of health. It reduced odds of preterm birth rates in Black participants, yet it is unclear which program components most contributed to this reduction. This study seeks to understand the experiences of staff and clients within the MAMA’s program to identify what factors decrease stress, how the program addresses racism and the challenges and opportunities of optimizing health during the COVID-19 pandemic.

**Methods:**

21 staff and 34 clients completed semi-structured interviews from November 2020–December 2021. Separate interview guides for staff and clients explored experiences within the program, experiences during the COVID-19 pandemic, and how racism affects clients. Interviews were recorded and transcribed. Analysis used a phenomenologic framework. Coding was performed using grounded theory to identify themes.

**Results:**

Analysis revealed six key themes: Stressors clients face, barriers for undocumented, Latina, and Spanish-speaking clients, exceptional care, emotional support, naming and responding to racism and discrimination, and impacts of COVID-19 pandemic.

**Discussion:**

Staff and clients work together to address social needs in order to address chronic stress and racism in their lives, especially during the COVID-19 pandemic. Interviews revealed relationship building is a cornerstone of the program’s success and plays a significant role in alleviating chronic stress in this population.

**Supplementary Information:**

The online version contains supplementary material available at 10.1007/s10995-024-03896-5.

## Significance

It is known that chronic stress is a risk factor for preterm birth. However, little is known about the experiences of pregnant women receiving social supports in addition to medical support. Prior studies have investigated experiences of perinatal women and accessing of resources during the COVID-19 pandemic, but no studies to date have evaluated the experiences in a comprehensive medical home that addresses both social determinants of health and social needs in addition to health care needs.

What this study adds: Perspectives of women at risk of preterm birth within a medical home that addresses both social and medical needs, perspectives of staff members, and an insight into what factors are deemed most impactful in reducing chronic stress, which may enable reduction in preterm birth.

**Supplementary Information:**

The online version contains supplementary material available at 10.1007/s10995-024-03896-5.

## Introduction

In the US, Black, Indigenous, and People of Color (BIPOC) birthing people experience inequities in birth outcomes which are influenced by social stressors, including social determinants of health (Manuck, 2017; Matoba et al., 2021; Rice et al., 2017). In 2021, non-Hispanic Black birthing people experienced preterm birth at higher rates than non-Hispanic White birthing people (14.75% and 9.5%, respectively) (Osterman et al., 2023). Drivers of the birth equity gap include social determinants of health such as secure housing, substance abuse, mental health disorders, and criminal-legal system involvement. These inequities may be shaped by systemic racism through mechanisms such as linguistic barriers, material hardship, lack of economic advancement, and lack of social supports (Rosenthal & Lobel, 2011; Wang et al., 2020). Furthermore, social stressors including discrimination and interpersonal racism contribute to preterm birth and adverse child health (Karvonen et al., 2022; Rosenthal & Lobel, 2011; Trent et al., 2019). Interventions are needed that address systemic racism and social determinants of health through care delivery innovation to improve perinatal health equity.

Acknowledging that experiences of adversity are complex and compounding, the Maternity Assessment and Management Access Service Synergy (MAMA’s) Neighborhood program was designed to address these challenges through client engagement with care coordination, extensive referrals, and leveraging cross-sectoral partnerships to meet clients’ needs through a client-centered approach. In Los Angeles (LA) County where 55% of women are Latina, 18.6% White, and 7% Black, MAMA’s is a medical home within the county’s safety-net healthcare system, in which the majority of birthing people are BIPOC, mostly Black^1^ or Latina^2^ (Los Angeles County Department of Public Health, 2016; Saleeby et al., 2021). MAMA’s is an integrated care model where health related social needs and mental health conditions are addressed by community health workers, health educators, social workers, and consultant psychologists/psychiatrists alongside standard evidence-based obstetric care provided by midwives, nurse practitioners, obstetricians, and maternal fetal medicine specialists.

A prior evaluation of the program demonstrated a 79% decrease in the adjusted odds of preterm birth among Black birthing people following program implementation in comparison to those who received standard care (Saleeby et al., 2021). Although MAMA’s was designed to address the stress that follows from racism, discrimination, and other social determinants of health, there are no studies exploring the potential mechanisms that drove down preterm birth rates.

The advantageous role of community health workers in promoting health and resiliency in underserved populations prior and during the COVID-19 pandemic has been described (Logan & Castañeda, 2020; Mayfield-Johnson et al., 2020). Gaining insight on what aspects of the program are deemed most effective in promoting health equity may reveal how successful perinatal health equity programs maintained their impacts during the pandemic, a time in which pregnant people experienced increased financial and social stressors and difficulties accessing services (Blebu et al., 2023). The purpose of this study is to understand staff and client perspectives regarding (1) how MAMA’s addresses chronic stress and health inequities among birthing people, including racism and discrimination and COVID-related stressors; and (2) how the COVID-19 pandemic affected MAMA’s.

## Methods

### Program Description

Enrollment in MAMA’s involves an in-depth psychosocial intake assessment by a Care Coordinator (community health worker) including stressors such as substance use, interpersonal violence, food insecurity, housing instability, social support, anxiety, depression, and corresponding assignment of a global stress score. The Care Coordinator then develops an individualized care plan with the client created to address unmet health related social needs (HRSNs) including active connection to substance use treatment programs, applications for housing or securing entry to shelters, community referrals to emergency food pantries, prenatal education and resiliency training, and referral to onsite mental health providers for evaluation and treatment, in addition to standard obstetric care. Intensity of care contacts for HRSNs are based on individualized stratification and those with highest stress scores are eligible to be contacted weekly while those with lowest stress once per trimester. Additionally, program staff host multidisciplinary meetings to troubleshoot barriers to care. Clients are followed for at least 18 months, and some qualified for home visitation (Saleeby et al., 2021).

### Research and Interviewer Team Composition

Five female researchers interviewed staff and clients (see Table 1 for interviewer details). The interview and analysis team came from White, Black, Latina, and Middle Eastern racial and ethnic backgrounds with professional backgrounds in public health and medicine. All interviewers were external to MAMA’s and had no contact with any participants outside of this study.

**Table 1:** Interviewer and analysis team characteristics

Interviewer	Title	Education and training	Ethnicity/race	Interview role	Analysis team
RD	Research Assistant	Bachelor of Arts	Hispanic/Latina	Staff Interviewer	No
SF	Research Coordinator	Master of Public Health	White	Staff Interviewer	No
FA	Research Assistant	Bachelor of Arts	Hispanic/Latina	Staff Interviewer; Spanish and English Client Interviewer	Yes
HIS	3rd Year Medical Student	Bachelor of Arts	Middle Eastern	Staff Interviewer; English Client Interviewer	Yes
KH	2nd year Neonatal-Perinatal Medicine Clinical Fellow	Bachelor of Arts, Doctor of Medicine, Master of Public Policy	Black/African American	English Client Interviewer	Yes

### Participant Selection and Recruitment

#### Staff Interviews

Purposeful sampling was used to recruit staff with diverse perspectives within MAMA’s and across LA County. To ensure participants had experience with the program prior to the pandemic, the sample was limited to those employed prior to March 2020, when the pandemic-related stay-at-home order was issued. Eligible staff were invited via email and those who responded and provided verbal consent via phone were enrolled. Semi-structured interviews in English were conducted via telephone or video conference and were recorded and transcribed. Staff participated voluntarily and did not receive compensation.

#### Client Interviews

Through purposeful sampling to include low and high-risk clients across LA County, MAMA’s staff supervisors identified and referred clients to the research team who invited their participation via text message or phone call. Those who provided oral informed consent were enrolled. Semi-structured interviews were conducted via telephone or video conference in English or Spanish and were recorded and transcribed. Clients were compensated $50 for 60 min of participation.

### Interview Guides

The semi-structured interview guides were developed from review of the literature involving social determinants of health, toxic stress, and birth outcomes, as well as discussions with MAMA’s leadership (Yardley, 2000). Separate interview guides were developed for staff and clients. Interview guides focused on (1) perceptions of effective program components and (2) challenges and opportunities for improving maternal health, generally, in relation to experiences of racism and discrimination, and in light of the COVID-19 pandemic (Online Appendix A). The client interview guide questions regarding racism and discrimination were posed to elicit answers specific to clients’ ethnicity and race, based on how clients self-identified.

### Analyses

Team members divided the transcripts and each reviewed roughly one third. Spanish transcripts were reviewed by the reviewer fluent in Spanish. Reviewers developed initial codes, which were combined to develop a universal codebook by consensus. Spanish interviews were then translated into English and all transcripts were coded in English by at least 2 reviewers using a phenomenological approach in Dedoose. Interviews, codebook development, and coding occurred in an iterative fashion until thematic saturation was achieved. Focused coding was completed using the constant comparative method to develop and refine themes. Staff interviews were completed from 11/9/2020–1/22/2021 and client interviews were conducted from 1/29/2021–2/1/2022. All procedures were performed in accord with prevailing ethical principles and were approved by the UCLA Institutional Review Board.

## Results

Twenty-one of 29 invited staff completed an interview (72%). Staff self-identified ethnicity and race and were able to select multiple races. Most staff participants identified as Latina (76%), few identified as Black (5%) and all identified as female (Table 2). Participants served in a wide variety of program roles and over half (52%) were employed for over 3 years.

**Table 2:** Interview participant characteristics

	Staff (%)	Clients (%)
N of respondents	21 (100)	34 (100)
Latino/Hispanic ethnicity	16 (76)	21 (62)
Primary language		
English	21 (100)	22 (65)
Spanish	–	12 (35)
Race		
Black/African American	1 (5)	8 (23)
White	8 (38)	17 (50)
Asian/Alaska Native/Pacific Islander	2 (10)	2 (6)
Declined to state race	1 (5)	2 (6)
Other	11 (52)	5 (15)
Age		
20–24	–	3 (9)
25–29	3 (14)	7 (20)
30–34	3 (14)	8 (23)
35–40	5 (24)	11 (33)
> 40	10 (48)	5 (15)
Highest level of education		
High school	1(5)	–
Associate’s	1(5)	–
Bachelor’s	7 (33)	–
Master’s	12 (57)	–
Role in MAMA’s		
Public health nurse	4 (19)	–
Nursing supervisor	1(5)	–
Clinical social worker	2 (10)	–
Psychiatric social worker	5 (24)	–
Community health worker/care coordinator	6 (29)	–
Senior care coordinator	1(5)	–
Health educator	2 (10)	–
Years working at MAMA’s		
0–1 years	6 (29)	–
1–2 years	4 (19)	–
3–4 years	8 (38)	–
> 4 years	3 (14)	–
Time as client in MAMA’s		
< 3 months		1 (3)
3–9 months	–	8 (24)
> 9 months	–	25 (73)
Receiving care for first pregnancy	–	23 (67)

Thirty-four of 61 invited clients completed an interview (56%).The majority of clients identified as Latina/Hispanic (62%) and 23% identified as Black, which is consistent with the demographic characteristics of MAMA’s clients in general, as described by Saleeby et al. (2021). Clients all identified as women and half self selected White race (50%), which included 40% who also identified as Latina. Participants mostly were receiving care for their first pregnancy (67%) and had been in the program for over 9 months (73%).

**Fig. 1:**
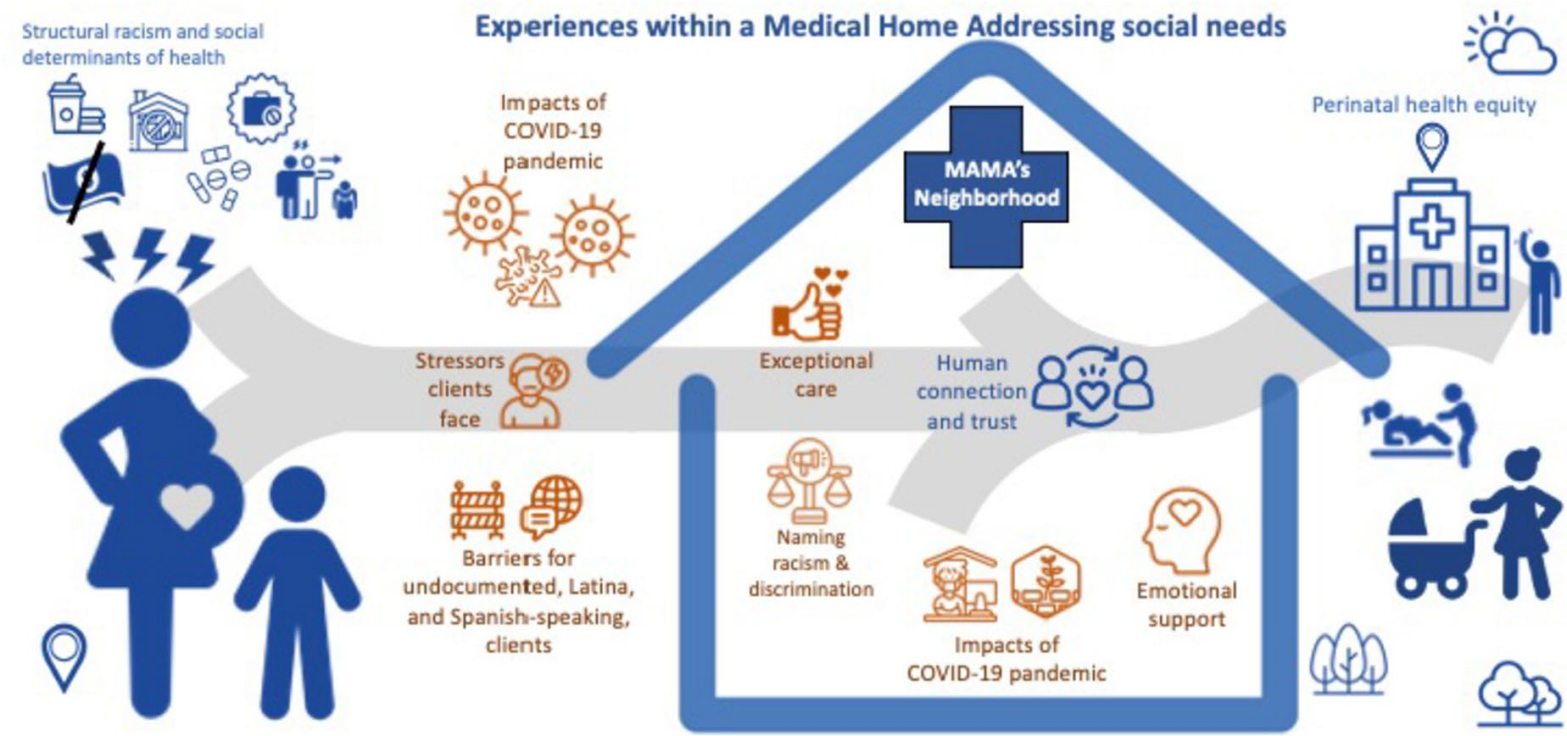
Patient experience in MAMA’s Neighborhood program. Clients in MAMA’s have stress related to structural racism and social determinants of health (SDOH) such as food insecurity, financial stress, housing and job insecurity, substance abuse, and racism and discrimination. This study’s six main themes are noted in orange, (1) stressors that clients face, noting an understanding of the SDOH related stressors that clients have before and during enrollment in MAMA’s, in addition to unique (2) barriers for undocumented, Latina, and Spanish-speaking clients. Within MAMA’s, staff and clients form trusting relationships based on human connection, influenced by (3) exceptional care and (4) emotional support. Staff and clients described experiences of unfair treatment within and outside of healthcare, noted by (5) naming and responding to racism and discrimination. Additionally, staff and clients described (6) impacts of the COVID-19 pandemic which heightened stress and affected client’s lives outside of the program, while affecting program execution. These themes describe how MAMA’s seeks to meet the aim of addressing chronic stress and achieving health equity in perinatal outcomes, or the equitable opportunity to realize one’s fullest potential. While this goal may not be fully realized, through the effort of MAMA’s, the system takes steps closer to realizing true perinatal health equity

Six main themes emerged from interviews, as noted in Table 3 and depicted in Fig. 1: (1) Stressors clients face, (2) Barriers for undocumented, Latina, and Spanish-speaking clients, (3) Exceptional care, (4) Emotional support, (5) Naming and Responding to Racism and Discrimination, and (6) Impacts of COVID-19 pandemic*.*

**Table 3:** Main themes and selected illustrative quotes

Theme	Subtheme	Description	Illustrative quotes
Stressors Clients Face	Material Hardships	Participants describe basic living needs and challenges related to social determinants of health	"We also have moms who are struggling with substance abuse. We try to link them into a program as soon as possible. The biggest challenge is that a lot of them don’t want to take clients who are pregnant… The question is where can we link them? " Staff participant 33, Hispanic or Latino, Psychiatric Social Worker, with MAMA’s over 4 years "Honestly, a lot of the time, they go back to jail, or into homelessness. When they go back to jail, sometimes we see they've delivered and their kids going to the [foster care] system. They don't have support.” Staff participant 18, Hispanic or Latino, Public Health Nurse, with MAMA’s 1–2 years “I fled from a situation of domestic violence. I was alone. I had nowhere to live. I didn’t even have a telephone. I had nothing. I [was put] in an emergency hotel and I did everything from a telephone. [MAMA’s] took me to apply for Medi-Cal so they could give me a phone. They helped me locate a shelter where I could stay and get psychological help. They started giving me classes to manage my pregnancy and diabetes. They guided me. I don’t know what I would have done if they hadn’t accepted the case.” Client participant 95 translated from Spanish, Hispanic or Latino, with MAMA’s over 9 months
	Emotional Stressors	Participants describe mental health needs	“I was under a lot of stress and anxiety. I was on psychiatric medication before my pregnancy… MAMA’s was very supportive while I was detoxing from the psychiatric meds. And I had rent issues because of [COVID-19]. I'm a recovering addict and alcoholic. I've been sober for four years. So, they were supportive all-around.” Client participant 67, Hispanic or Latino, with MAMA’s 3–9 months
Barriers for undocumented, Latina, and Spanish-Speaking Clients	Policy and Eligibility threats	Participants described unique barriers to care for undocumented clients, including immigration status	“[We] let them know and educate them about what would be a public charge, what wouldn't be, and what services are available to them and their rights… we make a lot of connections to the medical legal partnership we work with.” Staff participant 18, White, Hispanic or Latino, Community Health Worker, with MAMA’s 0-1 year “There's a big fear, for sure, for undocumented women that have Medi-Cal when I'm trying to get them to apply to primary care or to insurance other than emergency Medi-Cal. They won't do it. They're afraid of being a public charge. Even the women have gone as far as not wanting to take diapers” Staff participant 18, Hispanic or Latino, Public Health Nurse, with MAMA’s 3–4 years “I could have that kind of help, I did not want to do it… Even if they tell you that there is no problem, [and] everything is going to be confidential, I am really afraid of it..the program offered me a contact with some kind of lawyer who could help me with the documentation, but the fear is always there.” Client participant 87, translated from Spanish, Hispanic or Latino, with MAMA’s 2–4 years
	Language Barriers	Participants describe language differences and prolonged wait times	*In response to the question on how she's been treated differently because she's Latina, referring to her experiences with her previous clinic staff (outside of MAMA’s):* “They don’t pay much attention to you. I was talking, and they made fun of me. There was a guy there that spoke perfect Spanish and English, and another person there who only spoke English. It was like they would make me wait longer until one person could come along that could truly communicate with us.” Client participant 74, translated from Spanish, Hispanic or Latino, with MAMA’s over 9 months *The participants shares that when she needs transportation to the hospital, she calls MAMA’s staff. Her counselor explains that the participant does not speak English, but they still send drivers that do not speak Spanish. The drivers receive the hospital's general address, and they don't drop off in the right location:* “When I ask for transportation, the guys would only speak English and I sometimes do not know the address to the general hospital. They’d sometimes drop me off in the back… I tell him, ‘I don’t know. It’s not here.’ But the man only spoke English. I’m not going to go around with the heat and the baby.” Client participant 69 translated from Spanish, Hispanic or Latino, with MAMA’s over 9 months
	Embarrassment and Shame	Participants share feelings of embarrassment or shame when seeking aid	“A lot of people are like ‘I’ll do it myself. I can make it myself,’ but they [should] feel comfortable in asking and not being looked down upon or feel shamed that they need that extra help.” Staff participant 41, Hispanic or Latino, Health Education Assistant, with MAMA’s over 4 years
Exceptional Care		Staff work with a “whatever it takes” attitude and find creative ways to support clients even if outside of the anticipated scope of their work. Clients endorse that staff anticipate their needs and empower them to be self-reliant	“That's why I always ask, ‘Do you want me to send you a text or you want me to send you a picture?’ Because I have clients that tell me, ‘Oh, I don't know how to read.’ Fine, I’ll take pictures [of] how to do it step by step and I stick with them until they do it.” Staff participant 29, Hispanic or Latino, Community Health Worker, with MAMA’s 1–2 years “I actually got on three-way with her to call the clinic and help her make that appointment. Because some [clients] feel intimidated… I always ask, ‘Do you need my support? Do you need me to call with you? We could call together, or I could give you the number, whatever makes you feel more comfortable?’ And if they say, ‘Yeah, can you call with me?’ ‘Okay, let's do it. Let's call.’” Staff participant 19, Hispanic or Latino, Psychiatric Social Worker, with MAMA’s 0-1 year “I also like the fact that they were willing to help the father of my child, too, because there are not very many programs out there that are for dads, but MAMA’s program was ready to look for different programs that he can attend to.” Client Participant 53, Black/African American, with MAMA’s over 9 months “They offered pretty much everything that I didn't have or didn't even think of buying when it came to the baby. And they offered housing and job search, and just different groups and programs that I could join that would help me take care of my daughter.” Client participant 77, Black/African American, with MAMA’s over 9 months “With all my friends that are pregnant, I always give them the MAMA’s number, or my therapist [number] and be like ‘Reach out to her. She’s amazing.’ They’re just awesome.” Client Participant 44, Not Hispanic or Latino, Other: Mixed, with MAMA’s over 9 months
Emotional Support		The MAMA’s team strategizes to establish relationships and build rapport with their clients based on mutual respect. They empower clients to feel confident navigating medical care on their own. They address stigma related to mental health care and support connection to therapy and psychologists	“I’m here to support you, but when you get out of this program, I need you to be strong and know the system. I introduced the system to you, you navigate it, and I helped you navigate the system. And when you leave MAMA’s you’re ready to go.” Staff participant 40, Hispanic or Latino, Health Educator “I just know that there are a lot of clients who may not have a support system or someone to talk to on a personal level. So [we provide] someone to talk to where it's unbiased, non-judgmental, safe space. Even if they don't really say anything, just having that opportunity can be very meaningful to a lot of people.” Staff participant 25, Not Hispanic or Latino, Other: White and Black/African American, Community Health Worker, with MAMA’s 0–1 year “Sometimes we have some women that may smoke, and they'll cut down from smoking 15 cigarettes, a whole pack, to maybe just 2 and we at MAMA’s, we believe in harm reduction, so we celebrate that. Yeah, you're still exposing your child, but 15 to 2? That's huge.” -Staff participant 18, Hispanic/Latino, Public Health Nurse, with MAMA’s 3–4 years “I’ve always had somebody tell me what’s wrong with me. ‘You’re so dramatic or you’re too emotional, you’re too sensitive.’ She didn’t do any of that. She just told me everything you’re feeling is okay and it’s normal and any of the bad stuff that you don’t like feeling, we’re going to help you through it.” Client participant 107, Not Hispanic or Latino, White, with MAMA’s over 9 months “I was given a lot of advice, different options and moral support, spiritual support. It was kind of like having a therapist and a grandparent and a parent and a sibling -all of that wrapped in one person. And it was very helpful for me because I don't really have family support like that.” Client Participant 79, Not Hispanic or Latino, Black/African American, with MAMA’s over 9 months “And as soon as I said something like ‘I'm going through it but I'm staying strong. I just went through something with my husband’ the lady was like, ‘You know what? I think it would be great help for you to try to go to our counseling. I would love to refer you to one of our counselors.’" Client participant 98, Hispanic or Latino, with MAMA’s over 9 months “I do not feel like I would have made it through [pregnancy]. I mean I felt like I would have just lost my baby for how depressed I was and how much stress I was under. I had no hope… Honestly, I’m grateful for the help that they gave me and that I’m not in that state of mind that I was in.” Client Participant 81, Hispanic or Latino, with MAMA’s over 9 months
Naming and Responding to Racism and Discrimination	Trauma Informed Care Grounded in Knowledge of Racism	Staff share understanding of the historical context of racism within the US, the influences on health outcomes, and the development of medical mistrust	“Yeah, I think, unfortunately, history has shown women of color, People of Color, that maybe they’re just not welcome there, that they’re not believed. I know a big issue is People of Color don’t feel pain…there’s a history of these incidences where it’s now caused mistrust in the healthcare system. I believe it’s getting better. I think that’s why programs like us really are important, and it’s important to have that consistency so that they could see that there are people in the healthcare system that care and hopefully improve the trust for them and later generations.” Staff participant 27, Hispanic or Latino, White, Community Health Worker, with MAMA’s 0–1 year “[I] reassure the client…they have that choice to be vocal [and] to be heard, you have the right no matter what color you are…if you are feeling a certain way, the feeling that you're being discriminated [against] or if you feel like you are being mistreated in any way just to speak up, voice yourself.” Staff participant 21, Mixed, Community Health Worker, with MAMA’s 3–4 years “Yeah, I’ve heard a lot of stories of clients saying that they were complaining of pain or complaining of certain symptoms, and they’re not taken seriously.” Staff participant 45, Hispanic or Latino, Other: mixed, Public Health Nurse, with MAMA’s 3–4 years “I believe that racism affects our clients’ daily lives, depending on what their race is. If they are non-Latino, if they are African American, if they are [White], racism has clearly different impacts on their social status, where they live, their housing, availability, educational opportunities.” Staff participant 49, Not Hispanic or Latino, Black/African American, Community Health Worker, with MAMA’s 3–4 years
	Daily Accounts of Racism	Anecdotes depict experiences of discriminatory and racist treatment in daily life	“One patient mentioned that it happened at the supermarket. She was looking around to see what she was going to buy, and there was this young man following her from aisle to aisle. She said ‘He didn't know that I'd been feeling sick, that it was really hard for me to get up just to come to the market and I'm not eating really well because of my nausea. He didn't know that, but he was just there looking, assuming that I was going to take something’” Staff participant 41, Hispanic or Latino, Health Educator
	Naming Racism and Discrimination and Racial Concordance	Staff and clients identify instances of discrimination and racism. Staff look to advocate for clients when able. Participants discuss the role of racial concordance within the program	“She was, you know, a young Hispanic mom [with] this attitude of ‘you're not really going to do anything about it.’ Like, you know, she just, that was like her perception that that she thought she was being dismissed. Like there's just so many layers, and, you know, examples of that. And I always wonder like, yeah, like if spoke with her, or if I go there [as] like an older white woman? Like, would the response be, you know, different and easier, and it’s just messed up.” Staff participant 46, Not Hispanic or Latino, White, with MAMA’s 3–4 years “[We] make sure patients know what symptoms are normal and what aren’t. We don't let [anyone] dismiss them [or] say you're ‘this demographic, you just want a free hospital stay.’ [We want them to be] able to put their foot down and say, ‘No, I think something's wrong, and you need to run this test.’” Staff participant 25, Not Hispanic or Latino, White, Black/African American, Community Health Worker “I felt a lot of race and racism affecting the program. They brought in a lot of different African American women. And there were no African American staff members. And it's like, I understand you guys want to help us, but I felt like we needed somebody who we could relate to as people. I don't feel like a White woman could tell me, ‘Oh, yeah, I've been in your shoes before I understand what you're saying.’ It’s like, not really.” Client participant 79, Not Hispanic or Latino, Black/African American, with MAMA’s over 9 months
Impacts of COVID-19 Pandemic	Acute on Chronic Stress	Clients experienced increase in depression, anxiety, and fear of COVID-19 exposure. Yet, some benefitted from space to take time for themselves and practice self-care	“When you have little [money], it teaches you how to spend wisely. And you learn that you don't have to have it if you don't need it. Also [COVID-19] taught me patience. I'm able to be home during my pregnancy, relax and just focus on myself until the baby [gets] here.” Client participant 85, Not Hispanic or Latino, Black/African American, with MAMA’s over 9 months “There were signs on these like stores and the buildings that said, ‘Please, do not enter and please do not knock, we will not answer.’ …if it wasn’t for like a social worker helping and guiding and being determined, I don't think we would have gotten the help we needed.” Client participant 107, Not Hispanic or Latino, White, with MAMA’s over 9 months
	Rapport Building	Staff felt COVID-19 impacted interpersonal connections, which were hindered by loss of face-to-face interaction	“I think that trust is a little bit less there when we’re not in their space. When we [do home visits], we are not only building more trust with them, but we also get an idea of how they’re living, so we catch things like what can be a hazard for the baby…That’s what we can’t do now because we don’t have access to their home.” Staff participant 18, Hispanic or Latino, Public Health Nurse, with MAMA’s 3–4 years “Our clients genuinely appreciate the help they receive…it's just that reassurance that they're great moms…I think the client continues to benefit from it even though we're in the pandemic phase of client care.” Staff participant 49, Not Hispanic or Latino, Black/African American, Community Health Worker, with MAMA’s 3–4 months

### Theme 1: Stressors Clients Face

“I think this person is pregnant, she should be eating nutritious food. She should not have to have [stress] about diapers or ‘if I’m going to have enough food for the next week, or if my partner’s going to lose his job, or if I’m going to make it to pay the rent.’”

Staff participant 41, Hispanic or Latino, Health Educator

In addition to the stress of pregnancy, staff and clients reported that material hardships and other social stressors were identified and addressed by the program such as housing and food insecurity, substance abuse, domestic violence, job loss, and incarceration through community referrals to various programs i.e. shelters, rehab programs, WIC, etc.

Staff noted that clients’ depression and anxiety affect their relationships, and for some, their ability to perform daily tasks. A third of staff reported clients did not have access to mental health providers prior to enrollment in MAMA’s. Staff also described the challenges in coordinating medical care for clients in a system that poorly adapts to the instability in clients’ lives.

### Theme 2: Barriers for undocumented, Latina, and Spanish-Speaking Clients

Staff and clients described systemic barriers to care that disadvantage those who have immigrated to the US and who do not possess citizenship or legal residence status. Undocumented clients often lose their Medicaid insurance after giving birth, and fear of deportation was commonly cited as a reason for not seeking care despite referrals to legal services.

“It’s not uncommon to hear those questions of ‘Will this come back to me later? Will this affect my citizenship status?’ Yeah, there’s a lot of mistrust.”

Staff participant 27, Hispanic or Latino, White, Community Health Worker

MAMA’s staff and clients described how undocumented women face language barriers when accessing medical and supportive services or navigating transportation to appointments. A majority of Spanish-speaking clients described how language barriers influenced their interactions with medical providers and divulged how MAMA’s staff mediated three-way calls to help request translators and empowered them to utilize their right to interpreters. Latina clients in this study uniquely shared experiences of prolonged wait times, many in the setting of language barriers (e.g., waiting for an interpreter) and difficulty navigating transportation services. One client shared her experience in a medical visit:

“I was there an hour and a half. I went out and said, ‘Hey, what’s the delay? I’ve been here for too long.’ They then told me that they had forgotten and that they don’t speak Spanish...”

Client participant 74 translated from Spanish, Hispanic or Latino, with MAMA’s over 9 months

About a quarter of Latina clients shared that embarrassment or shame impeded them from seeking assistance from MAMA’s, regardless of encouragement from staff. One client expressed fear of “taking advantage” of the help that the program provided, and another client offered that having a menu of available resources for clients to choose from may help overcome shame associated with initiating the ask for help.

“If I tell them, maybe [MAMA’s] would help me, but I feel a little shame…because they help me with Pampers and to ask for more… it’s a shame.”

Client Participant 78 translated from Spanish, Hispanic or Latino, White, with MAMA’s 3–9 months

### Theme 3: Exceptional Care

Both clients and staff often referred to MAMA’s staff members as going “above and beyond” to address their needs. Participants shared staff exhibited a range of qualities, including cultural sensitivity, language concordance, and resourcefulness, all motivated by concern for clients’ wellbeing. Staff expressed they stay at the client’s side at every step, responding positively to client progress, even the completion of small action items. Over half of staff reported assisting with healthcare system and social service navigation such as making medical appointments and accessing WIC. Acknowledging the importance of self-reliance, over half of staff cited importance of avoiding paternalism and goal of teaching clients how to navigate the healthcare system so they can continue to do so independently after leaving the program. Clients praised the staff’s ability to anticipate their needs and offer services that were not known to clients and shared unanimous gratitude for the receipt of tangible items such as diapers and baby carriers.

Negative feedback from clients was minimal and criticism primarily stemmed from instances in which clients had miscommunications with staff or misunderstandings of the program’s mission. Clients and staff noted variation in availability of referral services in different areas of the county, and few clients relayed dissatisfaction in not attending educational classes.

### Theme 4: Emotional Support

“I liked their consistency. They made sure that [when] they [called] to me, that I was available to speak to them. I liked that they don’t treat you like a client or just any patient, they made me feel valuable”

Client Participant 101, Hispanic or Latino, with MAMA’s over 9 months

Staff and clients shared through consistent check-ins and conversation, staff create a reliable safe space for clients to be heard that is not provided elsewhere in the clients’ lives, especially for clients who experienced social marginalization. Staff provide a listening ear, while addressing stigma related to mental health care and facilitating formal connection to therapy. A minority of clients shared they began therapy for the first time in their lives because of mental health referrals from staff.

About half of staff perceive that providing tangible help for clients, such as diapers, aids in developing relationship with clients that evolves to a deeper emotional connection and simultaneously aids mothers who expressed material hardships. Clients noted that the emotional support they received from MAMA’s was genuine and distinct from other programs. As one client described the relationships with staff felt like the familial support they were missing in their lives:

“I feel like MAMA’s is a program that's there for people who feel like they're alone… But MAMA’s is like, a second mom, hence the name MAMA’s...”

Client participant 77, Not Hispanic or Latino, Black/African American, with MAMA’s over 9 months

Staff noted a key goal of the program is to establish a supportive relationship and build rapport with each client. Staff respect that clients may be hesitant to divulge information, and that previous negative experiences with providers may impact clients’ ability to build trust with staff.

### Theme 5: Naming and Responding to Racism and Discrimination

“Because the way I wear my mask, the way I have my hair, they don’t know how to interact with me. I have a tan, but I have dreadlocks. They don’t know how to interact with me, so they’re more disrespectful, more rude, more distant.”

Client participant 44, Not Hispanic or Latino, Mixed race, with MAMAs over 9 months

Staff and clients describe experiences of discriminatory and racist treatment throughout their daily lives—in grocery stores, places of work, gas stations, etc. Likewise, staff and clients describe instances of racism in the healthcare system in which clients feel slighted or dismissed by medical staff (Jones, 2018).^3^ Generally, Black clients more clearly labeled experiences of differential treatment as racism in comparison to Latina clients. Most Latina participants initially declined experiencing racism or discrimination but described instances in which they felt they were treated poorly, which they ascribed to limited English proficiency rather than their ethnicity or race. A couple of clients and staff noted how racial and ethnic concordance between staff and clients positively influenced experiences of the program.

“... [the clients] realize [we're] making drop offs, and they say, ‘Oh, you're just like me, you look just like me.’”

Staff participant 17, Hispanic or Latino, White, Public Health Nurse

Clients did not describe participating in specific programming that addressed racism and discrimination. Black clients voiced desire to discuss racism as part of MAMA’s and suggested holding sessions to discuss the impact of racism on their health, and specifically with Black providers, noting the impact of racial concordance. A majority of staff acknowledge the role of racism and discrimination in client’s lives, sharing their beliefs that an understanding of historical racism is part of trauma-informed care. Staff noted clients may feel unwilling to ask for help due to aversion for fulfilling negative stereotypes, or may avoid seeking services in anticipation of discrimination. Staff viewed their role as a trustworthy liaison who may be the last thread in the fraught relationship between clients and medical providers. Staff cited the importance of addressing their own implicit biases and privileges that may affect their treatment of clients.

### Theme 6: Impacts of COVID-19 Pandemic

“[Life] changed drastically because like, when it comes to social, emotional… it feels like – it's like you're in jail, like, but in your own home”

Client participant 82, Hispanic or Latino, White, with MAMA’s over 9 months

Both staff and clients reported that the perks of receiving diapers and tangible supplies was sorely missed due to social distancing, and some staff found ways to provide these resources via contactless drop-offs. Staff and clients noted that the stressors of daily life were heightened during the pandemic, resulting in a time of acute-on-chronic stress. Increased food insecurity, housing loss, and job loss were commonly reported in client interviews. Most common, was fear of contracting COVID-19 virus. Clients also described stress and fear related to the lack of birthing support due to COVID-19 related visitation policies.

“[Having a phone] doesn’t mean that they have a smartphone with data, or unlimited data. So being part of a video call for an hour can definitely eat into that.”

Staff participant 51, Not Hispanic or Latino, White, with MAMA’s 0–1 year

Staff shared unique perspectives of strained capacity for relationship building, which relied on face-to-face communication. In-person interactions had provided organic opportunities to check-in on the mental health and needs of clients, and home visitation was cancelled, taking away an opportunity to gain insights into clients’ lives.

## Discussion

We sought to understand staff and client perspectives regarding how MAMA’s addresses chronic stress and perinatal birth inequities. We found that building trust and supportive relationships between staff and clients was viewed as foundational to the program’s success. These relationships facilitated connection to resources, and the execution of individualized care plans. The challenges to rapport-building posed by the COVID-19 pandemic highlighted the importance of face-to-face check-ins and providing tangible care items to developing personal relationships. Notably, staff provided an additional layer of social and emotional assistance for marginalized women with authenticity and empathy. Clients shared that the staff served as a reliable support, in the ways that a mother or family member may support a loved one.

We explored how addressing racism and discrimination is essential to the effectiveness of MAMA’s. It is notable that all staff received training in race and healthy equity and cultural competence as part of employment in MAMA’s. It is possible that this training along with their lived experiences influenced their ability to acknowledge and respond to instances of racism and discrimination. Clients noted the positive impact of racial concordance between clients and staff, which is an important aspect for further exploration.

Similar to previous study of prenatal Latina clients in which cultural sensitivity, language concordance, and citizenship status concerns were salient in patient-centered care, this study underscores the unique experiences of Latina, Spanish-speaking, and undocumented clients in navigating health care, and further attention and exploration should be given in the setting of seeking social services (Bergman & Connaughton, 2013). Fear of deportation and medicolegal concerns were also distinct in interviews within this group. Notably, stigma related to speaking Spanish and xenophobia may play a role in these experiences, and this study did not seek to disentangle this relationship. Further investigation into waiting times for all clients, particularly Latina and non-primarily English-speaking clients is warranted.

This study provides insight to how the program utilizes relationships to address chronic stress and social determinants of health. While providing tangible goods and community referrals is important, the program focuses on developing human connection and a holistic and anti-racist approach to client wellbeing. It is possible that this combination of human connection and comprehensive medical and social service connections led to the decrease in preterm birth previously described. If so, findings suggest the need for a fundamental re-evaluation of health equity interventions to focus beyond a transactional model of increasing access to services alone. In doing so, we may be better poised to interrupt the intergenerational impacts of racism and discrimination on birth and life course health outcomes.

### Limitations

We sought to capture a wide range of client and staff experiences with the program. No client participants described leaving the program and very few expressed dissatisfactions, suggesting participants may be more highly engaged in MAMA’s. Although participants were assured confidentiality, both client and staff participant responses may be influenced by social desirability bias. Further, clients with more negative experiences of the program may have chosen not to participate in this study. Despite interviewing a diverse group of participants, extrapolating our findings to other Black and Latina communities should be met with caution.

## Conclusion

The MAMA’s program seeks to alleviate health inequities by addressing chronic stress through facilitating connections to medical and social services in the context of authentic supportive relationships. These relationships are foundational to facilitating receipt of medical and social services, especially in times of heightened stress and exacerbated inequities like the COVID-19 pandemic. Future studies are necessary to further explore how addressing social determinants of health, racism and discrimination, and health related social needs may achieve the goal of perinatal health equity and reduction in preterm birth.

### Supplementary Information

Below is the link to the electronic supplementary material.

Supplementary file1 (DOCX 14 kb)

## Data Availability

N/A.

## Abbreviations

BIPOC: Black, Indigenous, and People of Color
HRSN: Health related social needs
LA: Los Angeles
MAMA’s: Maternity Assessment and Management Access Service Synergy through the Neighborhood
UCLA: University of California, Los Angeles
WIC: Special Supplemental Nutrition Program for Women, Infant, and Children

## Footnotes

1 The term Black refers to the African diaspora/those with African ancestry, including Africans and African Americans.

2 Latina refers to women from Mexico, Central America, and Spanish-speaking countries in South America and the Caribbean.

3 Dr. Camara Jones defines racism as “a system of structuring opportunity and assigning value based on the social interpretation of how one looks, that unfairly disadvantages some individuals and communities, unfairly advantages other individuals and communities, and saps the strength of the whole society through the waste of human resources.”

## Code Availability

N/A.
