# Supplementary material for: “MAMA’s is like a second mom:” Client and Staff Experiences in a Comprehensive Social Risk Care Management Program Within a Perinatal Medical Home
Source: Matern Child Health J. 2024 Jan 31;28(7):1198–209. doi: 10.1007/s10995-024-03896-5 (PMC11180014; doi:10.1007/s10995-024-03896-5)
Supplement: Supplementary file 1 — Supplementary file1 (DOCX 14 kb) [file 10995_2024_3896_MOESM1_ESM.docx]

*Appendix A. Semi-Structured Interview Guide Sample Questions*

1. Tell me about your experience within the MAMA’s program.
   1. For Staff: What do you feel are the most important aspects of the MAMA’s program?
2. What comes to mind when you think of how your life has changed since the COVID-19 pandemic began?
   1. For staff: How has your work has changed since the COVID-19 pandemic began?
3. How has the COVID-19 pandemic affected your ability to manage your health?
   1. For staff: How has COVID-19 pandemic affected your clients’ ability to manage their health?
4. Have you ever been treated differently because of your ethnicity/race?
   1. For staff: How do you think that racism affects MAMA’s clients?
5. Have you been treated differently by doctors, nurses, or anyone else providing your medical care?
   1. For staff: Where do clients experience racism?
6. In what ways has your experience of COVID-19 been related to your ethnicity/Race?
   1. How has COVID-19 affected the racism that MAMA’s clients experience?
7. For staff: How do you think that the MAMA’s program can help clients cope with experiences of racism, discrimination, and the related stress?
